# Polyparasitism with Malaria and Intestinal Parasite Infections among Infants and Preschool-Aged Children in Egbedore, Osun State, Nigeria

**DOI:** 10.1155/2020/8810148

**Published:** 2020-07-24

**Authors:** S. N. Odoemene, A. S. Oluwole, H. O. Mogaji, M. V. Adegbola, O. O. Omitola, A. A. Bayegun, D. A. Ojo, S. O. Sam-Wobo, U. F. Ekpo

**Affiliations:** ^1^Department of Basic Sciences, Adeleke University, Ede, Osun State, Nigeria; ^2^Department of Pure and Applied Zoology, Federal University of Agriculture, Abeokuta, Nigeria; ^3^The COUNTDOWN Project, Department of Neglected Tropical Diseases, Sightsavers, Nigeria Country Office, Kaduna, Nigeria; ^4^Department of Animal and Environmental Biology, Federal University Oye-Ekiti, Ekiti State, Nigeria; ^5^Department of Science Laboratory Technology, Federal Polytechnics Ede, Nigeria; ^6^Department of Microbiology, Federal University of Agriculture, Abeokuta, Nigeria

## Abstract

Polyparasitism is widespread in many communities in Sub-Saharan Africa. However, there is paucity of data on polyparasitism in infants and preschool-aged children (IPSAC), to inform policy developments. Therefore, a survey of 1110 consented IPSAC was undertaken in Egbedore Local Government Area (LGA), Osun State, Nigeria, to determine the prevalence of polyparasitism in IPSAC in ten randomly selected rural communities. Fresh stool and blood samples were collected and processed for intestinal parasites and malaria infection. Mothers/caregivers were interviewed using a structured questionnaire to obtain demographic data of their IPSAC and to document knowledge, attitude, and practice (KAP) on parasitic infections. Data obtained through the questionnaire were analyzed using EpiData version 3.1, while parasitological data were analyzed using Statistical Package for Social Sciences (version 20.0). Descriptive statistics were computed for demographic data and association which were tested using bivariate analysis at a 95% confidence level while significance was set at *p* < 0.05. The results showed that 349 (46.29%) were infected with a single parasite. Infants and preschool-aged children infected with double, triple, and quadruple parasites are 268 (35.54%), 122 (16.18%), and 15 (1.99%), respectively. The prevalence of polyparasitism is 405 (53.71%). Although females (54.07%) were more infected than males (45.93%), there was no significant difference (*p* > 0.05) observed. Significantly (*p* < 0.05) more preschool children (65.93%) harbour more infections than the infants do (34.07%). Ara community (14.81%) had the highest cases of polyparasitized IPSAC, but no significant difference (*p* > 0.05) was observed across the communities. Double parasitic infection of *Plasmodium falciparum* and *Ascaris lumbricoides* (30.12%) and triple parasitic infection of *P. falciparum*, *A. lumbricoides*, and *T. trichiura* (14.81%) were the most common forms of polyparasitism encountered in the study. This study showed that polyparasitism is a burden in IPSAC and needs further investigation.

## 1. Introduction

Polyparasitism is widespread in many communities in the tropics and subtropics. Coinfection of malaria and intestinal parasites is among the most common polyparasitic infections in Sub-Saharan Africa (SSA), where significant proportions of the populations including infants and preschool-aged children, school-aged children, and adults are exposed to these infections [1, 2]. The most vulnerable group for these infections is children [3]. These diseases can affect a child's physical and mental development, educational achievements, social development, etc. There is a decrease in the prevalence of malaria infection in the world due to an increase in funding against the disease. In 2017, 219 million cases of malaria and 435,000 deaths were reported in 87 countries [4]. Majority of this malaria cases were reported in infants, children under five years, pregnant women, and HIV/AIDS patients [4].

In the tropics, intestinal parasites constitute a major public health problem, as these areas are often characterised by all the conditions (such as humid climate, poor sanitary conditions, lack of clean portable water, and poor socioeconomic status) favouring transmission of these infections. Helminthiasis is a major cause of morbidity, especially in resource-limited settings [5]. The incidence of soil-transmitted helminths (STHs) is approximately 50% in developed countries and reaches up to 95% in developing countries, with Sub-Saharan Africa (SSA) having the highest burden of these infections [6, 7]. It is estimated that children or preschool age account for 10–20% of those infected with STHs [8]. The most common of the STH infections are *Ascaris lumbricoides* infections, *Trichuris trichiura* infections, and the hookworm infections caused by *Necator americanus* and *Ancylostoma duodenale*, and in less-developed countries, it is common that children are parasitized with more than one species at the same time, “with resultant impairments in physical, intellectual, and cognitive development” [9]. In addition, the parasites tend to affect the immune response of infected children [8]. The transmission is enhanced by poor socioeconomic conditions, poor personal hygiene, and poor disposal of human excreta [10, 11].

Malaria is thought to be responsible for 35% of mortality in children under the age of five, 25% of maternal mortality, and 60% of hospital admissions for children under five [12]. National guidelines currently in place for malaria control include indoor residual spraying (IRS) in selected urban districts (covering over 100,000 households and 4% of the population at risk), free distribution of long-lasting insecticide-treated nets (LLITNs) at neonatal consultations, and free artemisinin-based combination therapy (ACT) at public health facilities (with over four million doses delivered in 2007–08, enough to treat almost 70% of the reported cases) [12].

In Nigeria, these infections are a major public health problem among infants and preschool-aged children [13].

Children coinfected with these parasites have less than optimal development, have reduced learning and school achievements [14, 15], and have increased susceptibility to other infections [16–18]. Epidemiological studies indicate that individuals coinfected with more than one parasite species are at risk of increased morbidity [14–17] as well as at a risk of developing frequent and more severe disease due to interactions among the infecting parasite species [18–21].

Infants and preschoolers comprise about 10-20% of the 3.5 billion people living in malaria and intestinal parasite endemic areas, and the evidence on the prevalence of these infections among these groups and the infantile is emerging, with a lot of gap in Nigeria [13, 22, 23].

There is paucity of data on polyparasitism with malaria and intestinal parasites in IPSAC. Therefore, the present study was undertaken to determine the prevalence of malaria and IPI coinfection, on infants and preschoolers.

## 2. Materials and Methods

### 2.1. Study Area and Population

**Figure 1:**
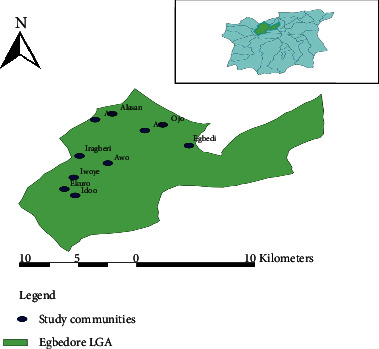
Map of Egbedore LGA showing the study areas.

Egbedore is one of 30 Local Government Areas (LGA) in Osun State, Southwest Nigeria. Its headquarters is in Awo town located at 7°46N and 4°24E. It has an area of 270 km^2^ with a population of 74,435 people as at 2006 census and projection of about 101,900 in 2016, out of which 21,231 were children between 0 and 5years. The main ethnic group is the Yorubas with Yoruba language as a means of communication. Majority of the inhabitants are engaged in small-scale farming; only few of the residents were civil servants. The inhabitants are mainly a Yoruba-speaking tribe that is made up of many towns, villages, districts, and 10 political wards headed by councilors. A Primary Health Center (PHC) is present in each of the communities. Data collection was carried out from May 2016 to February 2017 in ten rural communities (Figure 1).

### 2.2. Ethical Clearance

Approval for the study was obtained from Osun State Health Research Committee (OSHREC) Ministry of Health (Ref. Number: OSHREC/PRS/569T/66). The director of public health services in the LGA and coordinators of the Primary Health Center in each community sampled were notified about the study; they assisted in the sensitization and mobilization of mothers/caregivers. Informed consent forms were distributed to mothers/caregivers; the forms were verbally translated to the parents and caregiver in their local language; only mothers/caregivers who consented by signing the consent form were recruited into the study.

### 2.3. Study Design and Sampling Procedures

The survey employed a design involving the administration of a structured questionnaire for mother/caregivers and the collection of blood and stool samples from consented infants and preschoolers for laboratory analysis to check for malaria parasite and intestinal parasite infections. The inhabitants of the communities were sensitized prior to field surveys by the research team through their leaders, Community Development Association (CDA) meetings, Primary Health Care (PHC) staff, and community mobilizers. A total sampling of households within the communities was done, and consenting parents with children belonging to age category 0–6 years were recruited into the study in accordance with Ekpo et al. (2012).

Enumeration of households within the communities was done to enlist households with at least one or more infant and preschooler. Enumeration was followed simultaneously with recruitment of consenting parents/guardians and children (Figure 1).

### 2.4. Enrolment/Monitoring

Children whose parent consented to the study were enrolled into the study and given enrolment numbers (household ID) for easy identification and monitoring.

### 2.5. Data Collection and Laboratory Methods

#### 2.5.1. Stool Samples

Sterile universal bottle(s) were given to consenting parents/guardians for submission of their wards fresh stool samples. Eligible households were visited in the morning hours of the next day for collection of stool samples, which were later transported to the laboratory where assessment was carried out. Stool samples were analyzed using the sodium-acetate acetic-acid formalin-ether (SAF-ether) concentration method for the presence of intestinal parasite cysts, eggs, and ova. The SAF-ether method was employed to increase the sensitivity of detecting helminth and protozoan ova in stools as well as to preserve the sample prior the time of laboratory assessment. Samples were analyzed within 24 hours of collection.

Each bottle containing a stool sample was covered and agitated vigorously to efficiently suspend the stool in the solution. The stool suspension was strained into a centrifuge tube using double gauze of about 13 mm diameter placed in a funnel. The residue was discarded while the filtrate was centrifuged at 2000 rpm for 1 minute. The supernatant was discarded after centrifuging, and 7 ml of normal saline was added to the sediment and left to resuspend. 3 ml of ether was added to the suspension; a stopper was placed on the tube and the mixture shaken vigorously to mix before centrifuging for 5 minutes at 2000 rpm. The first three layers of the suspension observed after centrifuging was pipetted out using Pasteur's pipette leaving the last layer of sediment. The sediment was pipetted onto a clean, oil-free microscope slide and examined for ova of intestinal parasites using the Swiss TPH 2010 Diagnostic Bench Aids for species identifications using shapes of parasite eggs, cysts, and ova.

#### 2.5.2. Blood Samples

Blood samples were collected by hand pricking and examined microscopically for the presence of malaria parasites using malaria Rapid Diagnostic Tool (RDT). Infants and preschoolers were arranged in a census form and a corresponding number written on each test kit using permanent marker for identifications. The alcohol swab pack was used to clean the finger to be pricked using a circular motion for 10 seconds; the swab was allowed to air dry and a lancet was used to prick the finger of the respondents for a drop of blood; the lancet was discarded into the biohazard-labelled sharps container. The capillary pipette provided in the test kit was used to collect 10 *μ*l of blood; the collected blood was added to the round well on the test kit cassettes and absorbed by the embedded pad.

#### 2.5.3. Questionnaire Administration

Parents/caregivers whose children provided adequate stool specimens were carefully interviewed using a closed-end structured questionnaire in their different households. Demographic data of parent/caregiver and each infant and preschool, household sanitary, and personal hygiene conditions were documented using the questionnaire. Knowledge about malaria and intestinal parasitic infection and its transmissions, knowledge about coinfections, and attitude and practice of mothers/caregivers on parasitic infections and treatments were also documented using the structured questionnaire.

### 2.6. Data Analysis

Data obtained from the questionnaire and parasitological investigations were entered into Microsoft Excel version and analyzed using IBM Statistical Package for Social Sciences (SPSS) version 20.0. Descriptive statistics such as proportion and percentages was computed for demographic data while associations and relationship between risk factors and disease outcome was tested using chi-square and regression analysis at 95% confidence level.

## 3. Results

### 3.1. Study Population

A total of 1110 IPSAC were recruited for the study; 1060 (95.50%) met the inclusion criteria by providing stool samples and blood specimens and 516(48.68%) were males and 544(51.32%) were females between the age 0 and 72 months (Table 1).

### 3.2. Prevalence of Malaria and Intestinal Parasite Infections

*Plasmodium falciparum* (61.89%) was the single most prevalent parasitic infection recorded among IPSAC across the communities. There were no significant difference (*p* > 0.005) observed in *Plasmodium falciparum* infection in the communities; 23.21%, 7.16%, 4.87%, 0.29%, and 1.45% for *A. lumbricoides*, *T. trichiura*, *E. histolytica*, *Taenia spp*, *Giardia lamblia*, and Hookworm spp, respectively, were the intestinal parasitic infections (IPIs) observed in this study. A significant difference (*p* < 0.005) was observed between malaria parasite infections and intestinal parasitic infection (Table 2). Infants and preschool-aged children were commonly infected with *Plasmodium falciparum* (61.89%) than soil-transmitted helminths (STH) and other protozoan parasites (38.11%).

### 3.3. Prevalence of Polyparasitism

A total of 405 IPSAC were infected with polyparasitism, 66.17%, 30.12%, and 3.70% for double, triple, and quadruple parasite infections, respectively. *Plasmodium falciparum* and *A. lumbricoides* (30.12%); *P. falciparum*, *A. lumbricoides*, and *T. trichiura* (14.81%); and *P. falciparum*, *A. lumbricoides*, *T. trichiura*, and *E. histolytica* are the most prevalent double, triple, and quadruple parasitic infections recorded in this survey. No significant difference (*p* > 0.005) was observed in polyparasitic infections across the communities, but a significant difference (*p* < 0.005) was observed between double and triple infections (Table 3). Coinfections of malaria parasites and STH were the common parasitic combinations recorded in this survey.

**Figure 2:**
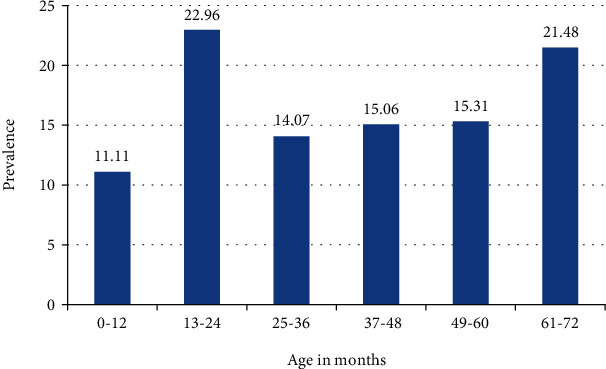
Prevalence of polyparasitism in relation to age of IPSAC.

A *t*-test between proportions was performed to determine whether there was a significant difference between single and polyparasitism by comparing their percentages across the communities. The *t*-test was significant at the 0.05 critical level *t* (753) = 2.043, *p* = 0.041 (Table 4). The prevalence of polyparasitism was higher in females, 219 (54.07%), compared to males, 186 (45.07%); however, no significant association (*p* = 0.340) was observed between polyparasitism and gender (Table 5). The prevalence of polyparasitism was higher in the IPSAC group aged 13-24 months (22.96%) compared to the IPSAC group aged 0-12 months (11.11%), but polyparasitic infections were more frequent in preschool-aged children (25-72 months) compared to infants (0-24 months) (65.93% vs. 34.07%, *p* = 0.001) (Figure 2).

### 3.4. Risk Factors of Polyparasitic Infections

**Figure 3:**
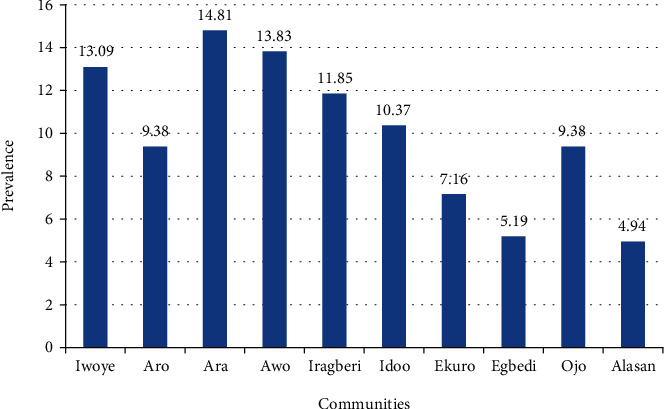
Prevalence of polyparasitism in the communities surveyed.

The risk factor of polyparasitic infection is shown in Table 5. There was a significant association between toilet facility and intestinal parasites; those who defecate in the bush or refuse dump were more likely to be infected with intestinal parasites (*χ*^2^ = 6.843, df = 3, *p* = 0.023) while ownership and utilization of LLIN is significantly associated (*p* < 0.005) with malaria infection; households that sleep regularly under the treated net had a low prevalence of malaria infection compared to those that did not. There were no associations between malaria infections, intestinal parasites, and risk factors such as water source, walking barefooted, causes of malaria or STH, deworming activities, and handwashing (Table 6). There was significant difference between educational status and polyparasitic infections in this study as IPSAC whose mothers/caregivers attended higher education had the lowest prevalence of polyparasitic infections. The highest polyparasitic infection was recorded in Ara community (14.81%) followed by 13.83% and 13.09% in Awo and Iwoye, respectively (Figure 3) while the least coinfection is recorded in Alasan (4.94%). However, there is no significant difference in the prevalence of coinfection in the communities (*p* > 0.230).

## 4. Discussions

Polyparasitism is widespread in rural communities in Sub-Saharan Africa where a significant proportion of people are exposed to coinfections of more than one parasite. Polyparasitic infections are more prevalent in the rural communities and are closely associated with poverty [24] and poor water sanitation and hygiene (WASH) conditions of rural communities [25]. Understanding the epidemiology of these infections within the community is essential in the design and implementation of control strategies by policymakers. The result of this cross-sectional survey shows a prevalence of 53.71% of polyparasitic infection in the study communities which is high compared to the findings of [26] where 38% of the children were infected with two or more parasites and [27] in coastal Kenya in which a prevalence of 31.80% was also recorded; the prevalence of polyparasitism in this present study is high which is an indication that polyparasitism is endemic in the study area; this could be due to favourable environmental factors such climate, adequate soil moisture, warm temperature for larva development, poverty, and improper hygiene condition which favour the transmission of the parasites.

According to [19], parasitic infection like malaria is directly related to the environmental situation observed in an area which promotes the transmission of malaria especially during rainy season. This study was carried out during the raining season; this might be responsible for high prevalence of malaria and intestinal parasitic infection cases recorded in this study, but other factors may include low socioeconomic status, ignorance, and poor sanitation. Similar findings were reported among school children in Côte d'Ivoire where children were typically infected with an average of two or more species concurrently [28]. However, there is no significant difference in the prevalence of coinfection recorded in the communities (*p* > 0.230).

The risk of polyparasitism increased with age; older children (25-72 months) were more affected compared to younger children (0-24 months). The differences were statistically significant by bivariate and multivariate regression analyses (*p* < 0.001). This reflects the exposure patterns in view of the fact they are more active, adventurous, and mindless of hygienic habits. Therefore, the infection rates in 0-24-month children were due to reduced exposure to the infective stages of the parasite as a result of less active adventures. The prevalence of polyparasitism was observed to be higher in females than in males in the study area which was statistically insignificant. The difference appears to confirm the possible roles of female activities that enhance relative contact with the infective stages of the parasites.

In Nigeria, a control program against helminth has been implemented through an expanded program using mebendazole or albendazole among school-aged children and the adult population; infants and preschool-aged children do not often benefit from this intervention which is provided only to school-aged children. However, infants and preschoolers have been targeted by interventions against malaria through the distribution of LLIN and antenatal and postnatal programs.

In conclusion, IPSAC in the study area are at considerable risk of polyparasitic infection with malaria, soil-transmitted helminths, and intestinal protozoa. Hence, measures are needed to combat the future occurrence of polyparasitic infections in this area in order to prevent IPSAC from infection with pathogenic helminths, intestinal protozoa, and malaria. The measures are proper personal hygiene, health education for mother/caregivers, improved access to clean drinking water and sanitation (good waste disposal system, provision of toilet facilities), use of mosquito nets, and proper treatment of infected persons in the communities; this measure should be promoted and advocated by government and stakeholders in the health sector.

Infants and preschoolers represent a reservoir for intestinal helminths and protozoan parasitic infections; therefore, routine screening for these infections in health facilities should be included as part of their health care packages.

## Figures and Tables

**Table 1:** Demographic status of infants and preschool-Aged children in the study communities.

Communities	Number of HH visited	IPSAC recruited	Samples collected	IPSAC infected (%)	Male (%)	Female (%)
Iwoye	48	140	133 (95.00)	90 (67.67)	58 (45.00)	75 (55.00)
Aro	44	115	112 (97.00)	66 (58.93)	55 (39.34)	57 (60.66)
Ara	50	130	120 (92.31)	89 (74.17)	56 (59.04)	64 (40.96)
Awo	52	166	156 (93.98)	120 (76.92)	76 (51.82)	80 (48.18)
Iragberi	40	108	108 (100)	81 (75.00)	61 (43.66)	47 (56.34)
Idoo	42	110	104 (94.55)	77 (74.03)	50 (43.28)	54 (56.72)
Ekuro	46	100	94 (94.00)	66 (70.21)	52 (57.16)	42 (42.85)
Edgedi	20	82	82 (100%)	52 (63.41)	39 (57.14)	43 (42.86)
Ojo	38	90	82 (91.11)	61 (74.39)	42 (45.10)	40 (54.90)
Alasan	25	69	69 (100)	47 (68.12)	27 (42.55)	42 (57.45)
Total	405	1110	1060 (95.50)	754 (71.13)	516 (48.68)	544 (51.32)

**Table 2:** Prevalence of single parasitic infections in the study area.

Parasites	Number infected	Percentage
*Plasmodium falciparum*	216	61.89
*Ascaris lumbricoides*	81	23.21
*Trichuris trichiura*	25	7.16
*Entamoeba histolytica*	17	4.87
Hookworm	4	1.15
*Taenia spp*	1	0.29
*Giardia lamblia*	5	1.43
Total	349	100

**Table 3:** Distribution pattern of polyparasites among infants and preschool children in the study communities.

Parasite combinations	Number infected	Percentage
Pf+As	122	30.12
As+Tt	48	11.85
Pf+Tt	37	9.14
Pf+Hw	9	2.22
Pf+Eh	11	2.72
As+Te	9	2.22
As+Hw	18	4.44
Pf+Te	3	0.74
Tt+Hw	5	1.23
As+Eh	5	1.23
Hw+Eh	1	0.25
Pf+As+Tt	60	14.81
As+Tt+Hw	10	2.47
Pf+As+Hw	26	6.42
As+Tt+Gl	6	1.48
Pf+As+Te	3	0.74
Pf+Tt+Eh	5	1.23
As+Tt+Eh	2	0.49
Pf+As+Eh	6	1.48
Tt+Hw+Te	4	0.99
Pf+As+Tt+Hw	4	0.99
Pf+As+Tt+Eh	11	2.72
Total	405	100

Mp: malaria parasite; As: *Ascaris lumbricoides*; Tt: *Trichuris trichiura*; Hw: hookworm; Eh: *Entamoeba histolytica*; Te: *Taenia spp*; Gl: *Giardia lamblia*.

**Table 4:** Association between single and polyparasitism profiles of IPSAC in the communities.

Communities	Infected	Single	Polyparasitism	*t*-test	Degree of freedom	*p* value
Iwoye	90	37 (41.11)	53 (58.89)	1.661	88	0.100
Aro	71	33 (46.48)	38 (53.52)	0.592	69	0.556
Ara	89	29 (32.58)	60 (67.42)	3.104	87	0.003
Awo	120	64 (53.33)	56 (46.67)	0.728	118	0.468
Iragberi	81	33 (40.74)	48 (59.26)	1.624	77	0.108
Idoo	77	35 (45.45)	42 (54.55)	0.795	75	0.429
Ekuro	66	37 (56.06)	29 (43.94)	0.977	64	0.332
Egbedi	52	31 (59.62)	21 (40.38)	1.362	50	0.179
Ojo	61	23 (37.70)	38 (62.30)	1.866	59	0.067
Alasan	47	27 (57.46)	20 (42.55)	1.011	45	0.317
Total	754	349 (46.30)	405 (53.70)	2.043	753	0.041

**Table 5:** Prevalence of polyparasitism by gender in infants and preschool-aged children in the study area.

Communities	Number examined	Polyparasitism (%)	Male (%)	Female (%)	*p* value
Iwoye	133	53 (33.83)	21 (40.00)	32 (60.00)	0.340
Aro	112	38 (36.61)	17 (48.78)	21 (51.22)	
Ara	120	60 (40.83)	26 (53.06)	34 (46.94)	
Awo	156	56 (35.90)	27 (48.21)	29 (51.79)	
Iragberi	108	48 (45.37)	26 (53.06)	22 (46.94)	
Idoo	104	42 (42.31)	19 (43.18)	23 (56.82)	
Ekuro	94	29 (46.81)	11 (38.64)	18 (61.36)	
Egbedi	82	21 (28.05)	9 (47.83)	12 (52.17)	
Ojo	82	38 (42.68)	17 (42.86)	21 (57.14)	
Alasan	69	20 (27.54)	13 (68.42)	07 (31.58)	
Total	1060	405 (100)	186 (45.93)	219 (54.07)	

**Table 6:** Association of polyparasitism with risk factors among study participants.

Variables	Polyparasitism	Percentage	*p* value
*Education*			
None	49	12.09	
Primary	181	44.69	
Secondary	152	37.53	
Higher education	23	5.68	0.012
*Toilet facility*			
None	340	83.95	
Pit	43	10.62	
Closet	22	5.43	0.023
*Water source*			
Tap	227	56.05	
Well	124	30.62	
Stream/river	10	2.47	0.145
*Deworming*			
Yes	273	67.41	
No	132	32.59	0.267
*LLIN usage*			
Yes	348	85.93	
No	57	14.07	0.002
*Handwashing*			
Regularly	365	90.12	
Not regularly	40	9.88	0.441
*Causes of worm*			
Eating of meat	159	39.26	
Sweet food	121	29.88	
Dirty	47	11.60	
Environment	78	19.26	0.115
Drug usage			
*Causes of malaria*			
Mosquito bite	356	87.90	
Sunlight	33	8.15	0.743
Oil consumption	16	3.95	
*Walk barefooted*			
Yes	381	94.07	
No	24	5.93	0.652

*χ* ^2^ = 6.843, *p* = 0.023.

## Data Availability

The dataset during and/or analyzed during the current study is available from the corresponding author on reasonable request.

## Abbreviations

IPSAC: Infant and preschool-aged children
LGA: Local Government Area
STH: Soil-transmitted helminths
RDT: Rapid Diagnostic Tool
CDA: Community Development Association
WHO: World Health Organization
PHC: Public health center
OSHREC: Osun State Health Research Committee.
